# Gonadal Hormone Changes with Aging and Their Impact on Chronic Pain

**DOI:** 10.3390/cells14020123

**Published:** 2025-01-16

**Authors:** Onella Athnaiel, Nicholas Davidson, Jaskaran Mangat, Ned F. Nasr, Nebojsa Nick Knezevic

**Affiliations:** 1 Advocate Illinois Masonic Medical Center, Department of Anesthesiology, Chicago, IL 60657, USA; onella.athnaiel@my.rfums.org (O.A.); nicholas.davidson@my.rfums.org (N.D.); jaskaran.mangat@aah.org (J.M.); ned.nasr@aah.org (N.F.N.); 2 Chicago Medical School, Rosalind Franklin University of Medicine and Science, North Chicago, IL 60064, USA; 3 Department of Anesthesiology, University of Illinois, Chicago, IL 60612, USA; 4 Department of Surgery, University of Illinois, Chicago, IL 60612, USA

**Keywords:** menopause, andropause, hormone changes, aging, pain perception, hormone replacement, chronic pain

## Abstract

Chronic pain, pain that lasts beyond three months, is a common finding in the elderly. It is often due to musculoskeletal conditions but can be precipitated by other factors as well. While physiological systems decline with aging, chronic pain is influenced by changes in hormone profiles as men and women enter into andropause and menopause, respectively. Research on gonadal hormones is limited, especially when it comes to their relationship with chronic pain. Women tend to experience less pain with aging compared to their premenopausal years, and this is partially explained by the fact that estrogen enhances pain sensitivity and its decline during menopause decreases it. However, hormone replacement therapy (HRT) seems to increase pain tolerance post-menopause. There is some evidence that testosterone plays a protective factor in pain perception. Men on the other hand, have higher pain tolerance as testosterone is considered to be a protective factor. With aging and decreasing testosterone, older men tend to be less tolerant to pain. This paper explores how hormonal changes with aging impact pain perception in both men and women, highlighting several pain conditions influenced by hormones. Although research remains limited, the potential of HRT as a treatment for common pain conditions is examined.

## 1. Introduction

According to the American Society of Anesthesiologists, chronic pain is defined as pain that does not go away, lasting beyond three months. Most pain goes away once treated, such as toothaches, sporadic headaches, or pain from injuries that eventually heal. Unlike the majority of pain, chronic pain lingers, which in turn affects the quality of life of millions of people around the world [1]. Chronic pain can have many triggers and causes which are discussed in more detail in this article. However, the long-term effects of chronic pain and the mechanisms that modulate this sensation are more consistent. There may be sex differences in the perception and tolerance of chronic pain which are explored here.

Approximately 21% of American adults live with chronic pain, which equates to about 51.6 million people. According to the Centers for Disease Control (CDC), more than 30.8% of adults aged 65 or older live with chronic pain, which is a higher proportion than all other age groups [2]. While the difference in the proportion of elderly men and women experiencing chronic pain is not clear, evidence suggests that women have a higher prevalence of chronic pain when compared to men (21.7% vs. 19.0%) [3]. A study comparing pain risk between men and women aged 63 or older followed the participants for 3 years. Results revealed that the risk of “highest pain” was 22.6% among women and 12.6% among men [4], consistent with the data found by the CDC.

Chronic pain is a widespread issue, particularly among the elderly, leading to functional disability and often co-occurring with psychological conditions like depression and anxiety, as well as cognitive and sleep disturbances. With the aging global population, managing chronic pain in older adults has become a significant clinical challenge. Common sources of pain in this group include neurodegenerative and musculoskeletal diseases, peripheral vascular conditions, arthritis, and osteoarthritis. These conditions contribute to poor quality of life, social isolation, decreased physical activity, and increased dependence on others for daily tasks [5].

The persistence of chronic pain is partly driven by central sensitization, a process involving synaptic plasticity and heightened neuronal responsiveness in central pain pathways following injury. This phenomenon is further influenced by neuroinflammation in the peripheral and central nervous systems. A hallmark of neuroinflammation is the activation of glial cells, such as microglia and astrocytes, within the spinal cord and brain, which release proinflammatory cytokines and chemokines, exacerbating pain [6]. It is worth noting that pain perception changes as an individual ages. Further, pain perception is different among males and females. Part of the reason is likely due to the hormone differences between the two and the way that males and females age [7]. The female hormone profiles are vastly different, and they remain different as they age. These differences lead to a different response to pain. Men and women suffer from different pain conditions likely due to hormone contributions and as the hormones change, pain conditions that begin prior to menopause tend to develop differently. In addition to pain perception differences, men and women tend to experience pain psychologically differently which may in turn affect their physiological responses as well [7,8,9]. Overall, pain perception is complex and has multiple factors, and such factors manifest in unique ways among men and women.

## 2. Basics of Pain Perception

Broadly speaking, pain perception is influenced by the biopsychosocial model [8]. The biological component involves the transmission of pain signals on a molecular level which, in turn, can be affected by hormones. The psychosocial factors can be more complex as they vary from person to person, but typically involve expectations, culture, social support, pre-existing psychological conditions, life experiences, upbringing, and many other factors that are often difficult to pinpoint and account for in clinical studies.

Pain originates when nociceptors, specialized nerve fibers in the skin, are activated by peripheral stimuli. These nociceptors contain molecular sensors in their nerve endings that detect various types of harmful stimuli and transmit this information to the spinal cord’s dorsal horn through the dorsal root ganglia. Nociceptors are associated with three main classes of nerve fibers—Aβ, Aδ, and C afferents—which are involved in pain signaling. These receptors are finely tuned to identify different types of noxious stimuli, such as mechanical, thermal, or chemical injuries, as well as immune mediators like bradykinin, cytokines, and histamine, along with ATP, microorganisms, and their toxins. The detected stimulus is then converted into an action potential, which is processed by the brain and interpreted as pain [5].

While some conflicting results exist, a review of existing studies suggests that estrogen plays a significant role in regulating pain by interacting with intracellular receptors, influencing gene expression, and modulating G-protein-coupled receptors in both the central and peripheral nervous systems. Furthermore, estrogen seems to affect various neural pathways involved in pain modulation, such as serotonergic, noradrenergic, dopaminergic, and particularly, the endogenous opioid systems. Normal levels of estrogen tend to activate the opioid system upon exposure to pain. While on the other hand, with decreased levels of estrogen, as in the menopause state, the system tends to be deactivated. Despite the impact of estrogen on opioid receptors, an endogenous pain-relieving system, estrogen is still generally believed to enhance pain sensitivity, and its effect has been well-documented in conditions like migraines, fibromyalgia, and tension-type headaches [9]. Fluctuations in estrogen levels enhance inflammation which in turn increase pain perception in females. In contrast to estrogen, progesterone has protective factors by increasing the expression of gamma-aminobutyric acid (GABA) in the brain [9].

The role of androgens in nociceptive pathways remains uncertain; however, the presence of androgen receptors in the limbic system and their ability to reduce estrogen receptor expression may explain some of the observed clinical differences in pain perception between men and women. Testosterone tends to deactivate activation of dorsal root ganglion in the spine. This suggests that androgens might offer a protective effect against nociceptive stimuli [9].

While hormonal changes are implicated in the perception of pain in both males and females, there are other factors that contribute to this complex sensation, especially in females. This includes the vasomotor symptoms associated with menopause, increased prevalence of musculoskeletal conditions, psychological symptoms, and sleep pattern changes. All these factors affect the perception of pain [10].

## 3. Effects of Aging on Pain Perception

Consistent with the mechanisms discussed previously, chronic pain is common among the elderly. The most common causes of pain in the elderly are a previous disorder, cancer, neuropathic pain, musculoskeletal, post-surgical, chronic headache, or chronic visceral pain [5]. Low back pain is very common among the elderly and one of the leading causes for seeking medical attention. Its prevalence ranges between 21–75%. Among those with low back pain, 60% of them have physical difficulties with daily activities. While the elderly tend to tolerate acute and sudden pain better than persistent pain, the mechanisms of pain transmission and perception in this population are still unclear. Many factors can play a role in this phenomenon, among them being hormonal changes and social support [11,12].

Studies have revealed that pain sensitivity may be decreased among the elderly [11]. Considering that pain has a functional effect of protecting against tissue damage and injury, this function may be compromised among the elderly and as a result, leads to more injuries which collectively may cause pain that is more difficult to treat. As opposed to acute pain, the elderly tend to be less tolerant towards persistent pain. This is most commonly seen in musculoskeletal pain. It is evident that pain perception changes with aging. However, it is also worth noting that much of this pain is related to other conditions and secondary factors [11,12].

**Figure 1:**
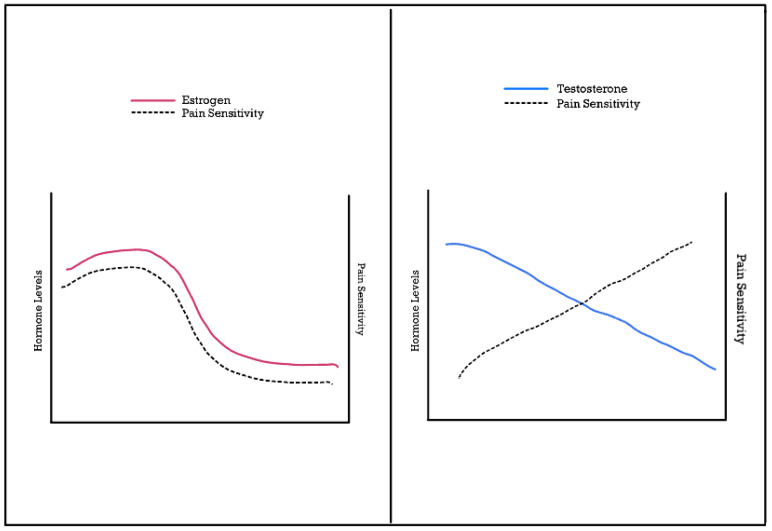
Sex hormones vs. pain sensitivity with aging.

When it comes to pain threshold, literature has consistently shown that with aging, the elderly tend to develop higher threshold levels of pain [12]. In other words, they tend to have less pain sensitivity as previously discussed, displayed in Figure 1. This is also shown in pain tolerance among the elderly. While their pain tolerance is higher when they approach 60 years old and beyond, this does not seem to be true at the higher levels of pain. In other words, once the pain reaches the upper limits, they tend to become less tolerant [13].

Despite the higher pain threshold among the elderly, it appears that their avoidant behaviors are diminished, especially in animal studies [12,14]. This entails that the emotional processing of pain may not be as robust as it is in younger rats. On the other hand, clinical studies have revealed that the elderly have a better sense of acceptance for pain in relation to younger individuals [12,15]. This may be attributed to many things, among which the helplessness and hopelessness that the elderly may develop after experiencing years of chronic pain as opposed to animal models that may not be the best representatives in this area.

## 4. Effect of Aging on Pain Perception in Females

According to the famous Framingham study, it appears that 50% of postmenopausal women suffer from osteoarthritis, one of the main causes of chronic pain in elderly women [16]. Another contributing factor to the increased musculoskeletal symptoms is the rapid reduction of bone density upon beginning of the menopause which often leads to osteopenia and later osteoporosis, both being associated with significant pain [10]. Women of reproductive age, despite having a lower threshold to pain compared to men, are protected against many of the musculoskeletal symptoms. As a result, such symptoms tend to appear abruptly as a woman transitions into pre- and post-menopause.

Aging significantly impacts pain perception in females, influenced by complex interactions between hormonal changes, neurobiological mechanisms, and psychosocial factors. Estrogen, a key hormone in females, plays a critical role in pain modulation. Premenopausal women during their reproductive ages tend to experience more pain compared to women, and the main reason for this response is the abrupt fluctuations in hormones throughout their menstrual cycle. For instance, the abrupt drop in estrogen mid cycle which is often followed by ovulation leads to the highest period of pain sensitivity in women [9]. As women age and transition into menopause, declining estrogen levels contribute to altered pain sensitivity. Studies indicate that premenopausal women generally experience enhanced pain sensitivity compared to men, potentially due to estrogen’s influence on pain pathways. The relationship between sex hormones and pain sensitivity is shown in Figure 1. This heightened sensitivity may decrease post-menopause, although older women still report a higher prevalence of chronic pain conditions compared to their male counterparts [7,9].

The relationship between estrogen and pain perception is mediated through its action on multiple neural pathways. Estrogen modulates nociceptive processing by interacting with serotonergic, noradrenergic, and opioid systems, which are involved in both the amplification and suppression of pain signals. These pathways also decline in efficacy with age, further complicating pain perception in older women. For example, postmenopausal women are at a higher risk of conditions like osteoarthritis, chronic migraines, and fibromyalgia—conditions linked to neuroinflammation and central sensitization. These processes are less efficiently modulated as hormonal levels decline. Considering the impact of estrogen on pain perception, the management of pain in females is also different. For instance, women tend to benefit from opioids more. At the same time, they tend to suffer from more side effects related to treatment [5,6]. The interaction between estrogen and pain management is not well defined; however, it certainly plays a role in how effective treatment is. More research needs to be done on the impact of estrogen levels and the response to medical management of pain.

Additionally, aging in females is accompanied by increased susceptibility to psychological comorbidities such as depression and anxiety, which exacerbate chronic pain. These comorbidities are particularly prevalent in older women, partly due to hormonal changes and life-stage transitions, such as retirement or caregiving roles, which influence their pain experience. Social support and cognitive resilience also play protective roles, mitigating the impact of pain in this population [12].

Although the reasons are yet unclear, one study investigating spinal cord stimulation in men and women aged >60 revealed that after one year of receiving treatment, post-menopausal women were more likely to improve on the pain scales compared to men [17]. It would be worth investigating whether the impact is similar in premenopausal women.

## 5. Effect of Aging on Pain Perception in Males

**Figure 2:**
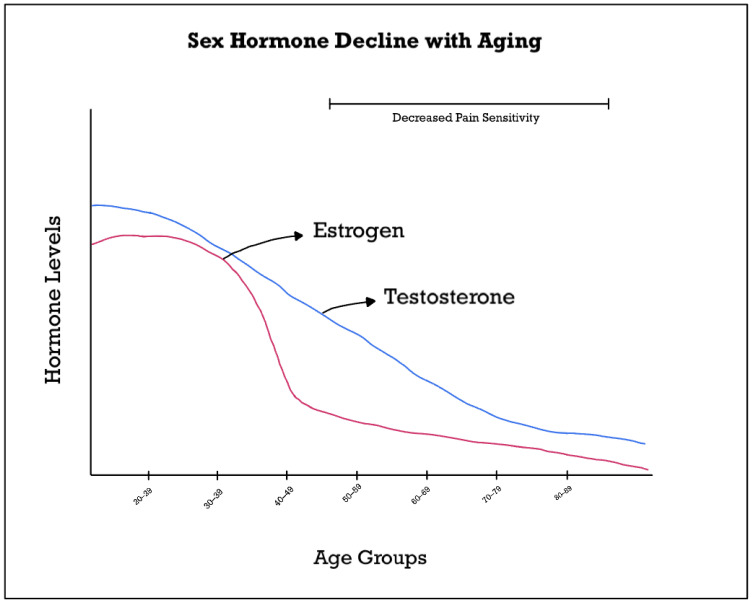
Trajectory of the main sex hormones with aging.

While there are insights into sex differences in pain perception across similar age-groups, there have not been many studies looking at specific age and gender interactions regarding pain perception. It is already well established that women experience chronic pain more than men, and differing levels of sex hormones as well as sexually dimorphic development could influence this [18]. As men age, their testosterone levels decline which can contribute to decreased bone mineral density, decreased muscle mass, and increased risk of cardiovascular disease [19]. In contrast to menopause in women, age related changes in testosterone (colloquially deemed “andropause”) happen gradually over time [20], displayed in Figure 2. Additionally, while menopause occurs in all females, not all males become testosterone deficient with aging [21]. Therefore, it may be harder to recognize the changes in physical health and pain perception that could be related to loss of testosterone because of these complexities [22].

Testosterone has been shown to influence pain perception [7,23,24,25]. Testosterone can affect the neuro-immune response to pain and modulate the expression of cannabinoid and mu-opioid receptors which have pain reducing effects, shown in Figure 1. A study by Lesnak et al. showed that testosterone provides protective factors to widespread musculoskeletal pain in mouse models regardless of sex [23]. Other studies have shown that testosterone can modulate pain perception through the descending pain modulatory system (DPMS), where higher levels of testosterone correlated with lower levels of unpleasant pain perception [21,25]. A recent study by Failla et al. showed that there could be age related changes in the activation of the DPMS between males and females, as well as greater activation of the insula and anterior cingulate cortex (ACC) leading to increased pain perception in older men after reaching a certain threshold [7].

The above shows an interesting connection between lower levels of testosterone and decreased pain tolerance, as well as how pain perception changes in aging males with decreased testosterone levels. Testosterone has been shown to be a protective factor against pain perception, and there is evidence for changes in the central pain pathways modulated by testosterone in aging males [7,23,24,25]. Men are also not exposed to as much pain throughout their lives as women [26]. Recent fMRI studies have shown that males have increased connectivity in brain areas associated with pain salience, and decreased activation of the DPMS compared to females. This could be associated with men being more attentive to their pain when present, as well as having less ability to habituate to pain [26]. It is possible that men do not undergo as much neuroplastic change in these areas due to less painful experiences throughout life, and this could contribute to greater pain perception in old age. In a similar manner, their response to pain treatment is not always consistent and it depends on multiple other factors such as life experiences, body composition, and other factors that influence physiological and psychological responses [27].

There are also many psychosocial and cultural factors that can influence pain reporting and perception which can confound study results [7,28]. Gender identity and social norms can influence pain reporting as well as the seeking of treatment for some conditions, with some studies showing a significant correlation between reported levels of masculinity and pain reporting in males [7,28]. Psychological factors like anxiety levels can influence pain reporting, with evidence showing higher anxiety levels positively correlated with higher pain reporting [28]. Additionally, the expectation of experiencing more pain with aging can possibly lead to less pain reporting. In many cultures, pain is considered part of the aging process and adults may hide their pain experiences because they feel a moral obligation [29]. Aging is a continuous process and since the human body is constantly evolving throughout this journey, one’s expectations and response to biological changes and pain are always changing. Depending on culture, people frame their pain from different lenses. For instance, in some cultures, going through crises is considered noble and a sign of strength. Such individuals are more likely to display their pain. On the other hand, some cultures take pride in having a positive attitude towards pain and such tend to minimize pain [29]. This complex and multifactorial experience can be hard to account for in studies of pain perception because despite similar experiences within similar groups, individuals are still different, and each person is unique in experiencing pain in their own way.

## 6. Chronic Pain Conditions Commonly Affected by Hormonal Changes

The following sections will explore how hormonal changes with aging affect these chronic pain conditions and whether hormone replacement can have an impact on the symptoms. The prevalence of these conditions in aging men and women is summarized in Table 1.

### 6.1. Migraines

Migraines are a debilitating form of headache influenced by hormonal changes, particularly fluctuations in estrogen. The prevalence of migraines is significantly higher in women than men, a disparity that becomes evident at puberty and persists through reproductive years. This is attributed to the role of estrogen in modulating neural excitability within the trigeminovascular system, a key pathway implicated in migraine pathophysiology [7]. Women with menstrual-related migraines often report that attacks occur just before or during menstruation, when estrogen levels drop. This hormonal sensitivity suggests that migraines are not solely triggered by external stimuli but are also heavily influenced by internal hormonal cycles [9].

Pregnancy and menopause further highlight the impact of hormonal changes on migraines. During pregnancy, particularly in the second and third trimesters, many women experience a reduction in migraine frequency due to stable and elevated estrogen levels. Conversely, menopause often exacerbates migraines due to erratic or declining estrogen levels, though some women report improvement, particularly in cases of non-menstrual migraines [6]. Hormone replacement therapy (HRT) used during menopause can variably influence migraine patterns, with some women experiencing relief while others report worsening symptoms, particularly with fluctuating estrogen doses [5].

In men, migraines are less prevalent, but hormonal influences still exist, albeit less understood. Testosterone’s potential modulatory role in neural pathways suggests a protective effect, further reinforcing the gender-specific differences in migraine presentation and severity [6]. This suggests that elderly men may be more likely to report migraine symptoms as testosterone levels drop with aging.

Older adults present unique challenges in the context of migraine management. While the prevalence of migraine generally declines with age, approximately 10% of older adults still experience migraines annually, with some studies indicating that elderly and disabled individuals report migraine burdens nearly equivalent to younger adults aged 18–44 years [30,31]. Contrary to earlier beliefs that menopause alleviates migraines, postmenopausal individuals may continue to experience significant symptoms due to persistent hormonal influences and the exacerbation of migraines by age-related comorbidities. Research indicates that individuals who first experienced migraines before the age of 18 are more likely to have persistent migraines in older age, with one-third of this population continuing to report migraines after age 64 [30]. Furthermore, age-related physiological changes, including slower gastric emptying, reduced hepatic mass and blood flow, and declining renal function, can significantly alter the pharmacokinetics and pharmacodynamics of migraine treatments, reducing therapeutic efficacy and increasing the risk of adverse effects [32,33,34]. These factors are compounded by a higher prevalence of comorbid conditions such as diabetes, hypertension, and cerebrovascular disease, which not only complicate treatment strategies but may also increase the risk of migraine chronification and disability if left untreated or poorly controlled [33]. Collectively, these findings underscore the need for an individualized and comprehensive approach to migraine management in older adults, considering both the physiological and comorbid complexity of this population.

### 6.2. Fibromyalgia

Fibromyalgia is a chronic pain syndrome characterized by widespread musculoskeletal pain, fatigue, sleep disturbances, and cognitive impairments. Women are disproportionately affected by fibromyalgia, and hormonal changes are believed to play a significant role in its pathophysiology. Dysregulation of estrogen and cortisol—key hormones involved in stress responses and pain modulation—has been implicated in heightened pain sensitivity and neuroinflammation in fibromyalgia patients [12]. Estrogen interacts with central pain processing pathways, particularly the serotonergic and endogenous opioid systems, amplifying nociceptive signals and contributing to the widespread pain experienced by individuals with fibromyalgia [13].

Aging compounds the effects of hormonal dysregulation. Postmenopausal women often report worsening fibromyalgia symptoms, a phenomenon associated with declining estrogen levels and diminished hypothalamic–pituitary–adrenal (HPA) axis responsiveness. Hormonal replacement therapies have shown mixed outcomes, with some studies indicating improved pain and fatigue, while others find minimal or no benefit [11]. Additionally, changes in central sensitization mechanisms with age, including reduced descending pain inhibitory control, exacerbate fibromyalgia symptoms in older adults [12].

Psychological factors such as stress, anxiety, and depression, which often co-occur with fibromyalgia, are also influenced by hormonal changes and aging, further complicating symptom management in this population [12].

### 6.3. Degenerative Changes

Degenerative changes, including osteoarthritis, osteoporosis, and intervertebral disc degeneration, are primary contributors to chronic pain and functional decline in older adults. These conditions are heavily influenced by hormonal changes, particularly the decline in estrogen and testosterone levels with aging. In a survey of menopausal women, more than half of them reported having back pain and joint stiffness which was similar to the prevalence of hot flashes [10,35].

Estrogen plays a critical role in maintaining joint and bone health by promoting cartilage integrity, reducing synovial inflammation, and preserving bone density. The drop in estrogen levels during menopause accelerates cartilage degradation and bone resorption, increasing the risk of osteoarthritis and osteoporosis in postmenopausal women [6]. In osteoarthritis, the loss of estrogen’s protective effects leads to heightened inflammatory responses within the joint space, further contributing to pain and mobility limitations [7].

Similarly, intervertebral disc degeneration—a major cause of chronic low back pain—is associated with age-related declines in anabolic signaling pathways, including those modulated by estrogen and testosterone. Testosterone, while less studied, contributes to musculoskeletal health by maintaining muscle mass and joint stability. Its gradual decline in men with aging results in reduced physical resilience, though the degenerative effects are generally less severe than those linked to estrogen in women [9].

Musculoskeletal pain (MSP) is a common complaint of perimenopausal and postmenopausal women, with an estimated prevalence of 71% among perimenopausal women [10,36]. A recent meta-analysis explored the relationship between menopause and the development of MSP in this population. The study showed that perimenopausal women were more likely to experience MSP than premenopausal women, and that postmenopausal women were more likely to experience more severe MSP symptoms than both premenopausal and perimenopausal women. Additionally, the odds of developing severe MSP increased in a linear fashion from pre-menopause to post-menopause [36]. While the aforementioned effects of reduced estrogen on bone and joint health are likely contributors to the heightened pain experienced by peri- and postmenopausal women, this study shows that menopause may be an independent risk factor for the development of MSP. More studies are needed to address hormonal changes through the course of menopause and their relation to MSP development.

Management strategies for degenerative conditions often include pharmacologic and non-pharmacologic interventions. Hormone replacement therapies, such as estrogen supplementation, have been explored for mitigating osteoarthritis and osteoporosis progression, though their use remains controversial due to potential cardiovascular and oncological risks [13].

### 6.4. Cluster Headaches in Males

Cluster headaches are a type of primary headache that is relatively rare when compared to tension and migraine headaches. They are typically seen in about 0.1% of the population. As a result, it is difficult to obtain data from such a small group [37].

Cluster headaches are more common in young males than any other age group, often diagnosed at 20–40 years of age. It is approximated that cluster headaches occur in a 2:1 ratio of men to women [38]. While the mean age at onset appears to be in the 30s for both males and females, when patients with chronic cluster headaches are considered, a bimodal distribution is more representative (2nd and 6th decades of life in women vs. 3rd and 5th decades of life in men) [39].

In a separate study investigating the prevalence of cluster headaches in different age groups, it appeared that with aging, prevalence of cluster headaches became more dominant in females compared to males especially in participants over the age of 65. In this group specifically, chronic cluster headaches were more common, rather than acute episodes [31]. Consistently, Mosek et al. reported that 15% of cluster headache diagnoses are made at age 50 or older in women. While the prevalence is at different ages among men and women, the characteristics of symptoms and their severity appears to be similar to symptoms that younger males experience [40].

A 2021 study comparing groups of men experiencing migraine and cluster headache with healthy headache controls found that men experiencing headache symptoms reported more symptoms of clinical androgen deficiency than did healthy controls [41]. There has been limited exploration of the effects of sex hormones on cluster headache and migraine in males. A small-scale study showed improvement in cluster headache symptoms after testosterone replacement therapy in some participants [42].

## 7. Hormone Replacement Therapy as Pain Treatment

Emerging evidence suggests that hormone replacement therapy (HRT) may help alleviate chronic pain in some postmenopausal women by restoring some of the pain-modulating effects of estrogen [11]. However, the efficacy and safety of HRT for pain management remain subjects of ongoing research. The benefits and risks of HRT are summarized in Table 2. In a separate study, the effects of resveratrol, a phytoestrogen, were investigated in 125 healthy postmenopausal women over the span of 24 months. The results revealed that pain scores and general well-being scores significantly improved, and results were noticeable after only 12 months of supplementation. The pain scores were obtained specifically in the context of musculoskeletal pain such as osteoarthritis, a very common cause of pain in elderly and postmenopausal women [43]. Understanding these gender-specific differences in pain perception and their underlying mechanisms is essential for developing targeted pain management strategies for older women.

One animal study investigating pain perception revealed that rats that had received ovariectomies had higher sensitivity to pain shown as increased muscle nociception. However, upon beginning with HRT, pain was reduced by 50%. This suggests that patients who have reached menopause, modeled by ovariectomies in rats, may benefit from hormone replacement especially when it comes to pain [44]. A similar study attempted to investigate the impact of HRT on pain responses in postmenopausal women. Results revealed that women in the HRT group had higher pain threshold and tolerance compared to their male counterparts and the women in the same age group not receiving HRT [45]. Results from both of these studies suggest that while premenopausal females tend to have lower pain thresholds compared to males, postmenopausal females are at an even higher risk of experiencing pain especially if not receiving HRT. While female sex hormones may be contributing to the lower pain tolerance, their lack in postmenopausal women does not get rid of the pain. Estrogen deficiency leads to many menopause symptoms such as hot flashes, vaginal dryness and vulvar pain, night sweats, and many other symptoms. Systemic and topical hormone therapy tends to relieve some of these symptoms with topical options being more effective for vulvar pain. However, HRT does not fully remove vulvar pain [46].

When it comes to testosterone replacement in men, research is limited due to the gradual decrease in testosterone with aging as opposed to the more abrupt drop in women. As a result, the symptoms tend to appear more gradually in males. Nevertheless, there are men receiving testosterone replacement, and literature has shown that it improves symptoms associated with hypogonadism and in return improves their quality of life significantly. There is also evidence that elderly men specifically receiving intramuscular testosterone showed the most improved muscle strength and increased lean body mass, particularly in the lower extremities [47,48]. This could be advantageous for elderly males because the loss of skeletal muscle mass in the lower extremities has been shown to be related to geriatric chronic pain [49]. On the other hand, testosterone replacement is associated with side effects such as increased serum viscosity which can lead to undesirable outcomes such as strokes, myocardial infarctions, and deep vein thrombosis [24]. Such evidence brings up a serious concern about the use of testosterone for symptoms associated with the decreased testosterone levels. There is also a gap in the research related to using HRT for pain management, and one should weigh the benefits and risks of the treatment before initiating this treatment in middle-aged and elderly men [50].

## 8. Conclusions

The perception of pain is a complex and multifactorial process. Hormonal changes associated with aging certainly play a role in pain sensitivity and tolerance. Women tend to have a lower pain threshold compared to men, which, in their premenopausal years, may be attributed to the cyclical nature of their sex hormones. Following the decline in hormone levels due to menopause, women often experience increased pain sensitivity, as the lack of estrogen is thought to enhance pain perception. Men, on the other hand, typically have a higher pain threshold due to the presence of testosterone. However, as testosterone levels gradually decrease with age, their pain sensitivity may increase. Despite these findings, pain is a complex phenomenon, influenced by factors beyond hormonal changes. Future studies could explore pain conditions exacerbated by hormonal fluctuations and investigate potential pain relief strategies, possibly through hormone replacement therapy.

## Figures and Tables

**Table 1:** Prevalence of pain conditions post-menopause/andropause.

Condition	Males	Females
Migraines	↑/-	↑ if menstrual-related migraine ↓ if not menstrual-related migraine
Fibromyalgia	↑ ^1^	↑
Degenerative Diseases	↑	↑
Cluster Headaches	↓	↑

^1^ Possibly underestimated in this population due to symptoms being attributed to other factors such as arthritis. ↑ increase; ↓ decrease; - unclear.

**Table 2:** Benefits and risks of hormone replacement therapy for pain management.

Benefits	Risks
Restoring hormones helps increase pain tolerance in postmenopausal women Helps minimize the other side effects associated with menopause in women, ie., hot flashes Testosterone replacement has been shown to restore lean muscle mass in elderly men which is protective against chronic pain and injury Testosterone replacement can help protect against widespread musculoskeletal pain and fibromyalgia	Unpredictable response for migraines in women as some benefit and some have worsening headaches Estrogen replacement may increase risk of deep vein thrombosis in women Possible adverse risks of testosterone replacement in men include serum hyperviscosity leading to CVA, MI, PE, and DVT Use of hormone therapy can have widespread effects on other body systems Research is limited and shows variable effects in both sexes
